# Diabetes-Induced Hepatic Pathogenic Damage, Inflammation, Oxidative Stress, and Insulin Resistance Was Exacerbated in Zinc Deficient Mouse Model

**DOI:** 10.1371/journal.pone.0049257

**Published:** 2012-12-12

**Authors:** Chi Zhang, Xuemian Lu, Yi Tan, Bing Li, Xiao Miao, Litai Jin, Xue Shi, Xiang Zhang, Lining Miao, Xiaokun Li, Lu Cai

**Affiliations:** 1 Ruian Center of the Chinese-American Research Institute for Diabetic Complications, The Third Affiliated Hospital of the Wenzhou Medical College, Wenzhou, Zhejiang, People's Republic of China; 2 The Chinese-American Research Institute for Diabetic Complications, The Wenzhou Medical College, Wenzhou, Zhejiang, People's Republic of China; 3 Kosair Children Hospital Research Institute, at the Department of Pediatrics of University of Louisville School of Medicine, Louisville, Kentucky, United States of America; 4 The Second Hospital of Jilin University, Changchun, People's Republic of China; 5 Department of Chemistry, University of Louisville, Louisville, Kentucky, United States of America; 6 Departments of Pharmacology and Toxicology, and Radiation Oncology, the University of Louisville, Louisville, Kentucky, United States of America; University of Hong Kong, China

## Abstract

**Objectives:**

Zinc (Zn) deficiency often occurs in the patients with diabetes. Effects of Zn deficiency on diabetes-induced hepatic injury were investigated.

**Methods:**

Type 1 diabetes was induced in FVB mice with multiple low-dose streptozotocin. Hyperglycemic and age-matched control mice were treated with and without Zn chelator, N,N,N′,N′-tetrakis (2-pyridylemethyl) ethylenediamine (TPEN), at 5 mg/kg body-weight daily for 4 months. Hepatic injury was examined by serum alanine aminotransferase (ALT) level and liver histopathological and biochemical changes.

**Results:**

Hepatic Zn deficiency (lower than control level, p<0.05) was seen in the mice with either diabetes or TPEN treatment and more evident in the mice with both diabetes and TPEN. Zn deficiency exacerbated hepatic injuries, shown by further increased serum ALT, hepatic lipid accumulation, inflammation, oxidative damage, and endoplasmic reticulum stress-related cell death in Diabetes/TPEN group compared to Diabetes alone. Diabetes/TPEN group also showed a significant decrease in nuclear factor-erythroid 2-related factor 2 (Nrf2) expression and transcription action along with significant increases in Akt negative regulators, decrease in Akt and GSK-3β phosphorylation, and increase in nuclear accumulation of Fyn (a Nrf2 negative regulator). *In vitro* study with HepG2 cells showed that apoptotic effect of TPEN at 0.5–1.0 µM could be completely prevented by simultaneous Zn supplementation at the dose range of 30–50 µM.

**Conclusions:**

Zn is required for maintaining Akt activation by inhibiting the expression of Akt negative regulators; Akt activation can inhibit Fyn nuclear translocation to export nuclear Nrf2 to cytoplasm for degradation. Zn deficiency significantly enhanced diabetes-induced hepatic injury likely through down-regulation of Nrf2 function.

## Introduction

Diabetes mellitus (DM) has become one of the most severe endocrine metabolic disorders in the world. Diabetes damages multiple organs to induce serious complications such as coronary artery disease, renal and ophthalmologic diseases that can result in the disability and mortality for diabetic patients. Liver disease as one of diabetic complications has not been well addressed, but it actually can be very significant [1]. Increasing evidence suggests that among patients with diabetes, the standardized mortality rate from end-stage liver disease (i.e., cirrhosis) is higher than that for cardiovascular disease [2], [3].

The liver plays a pivotal role in glucose homeostasis since it stores glycogen in the fed state and produces glucose through glycogenolysis and gluconeogenesis in the postabsorptive period. Several hormones and metabolic factors engage in the maintenance of glucose homeostasis. In physiological conditions, hepatocytes are the main site of hepatic glucose metabolism. It has been estimated that 30 to 60% of all glucose absorbed in the gastrointestinal tract undergoes hepatic processing with subsequent storage as glycogen or metabolism into amino acids or fatty acids [3], [4]. Insulin and glucagon are two counter-regulatory hormones involved in the regulation of energy metabolism. Insulin enhances glycogen synthesis within the liver and prevents glucose production. Reversely, glucagon induces glucose production and prevents glycogen synthesis [3]. The failure of hepatocytes to respond to insulin induced by diabetes results in uncontrolled gluconeogenesis, glycogenolysis and lipogenesis, promoting hyperglycemia, dyslipidemia and systemic insulin resistance [3]–[5], which will lead to diabetic liver complications such as steatohepatitis, chronic viral hepatitis, and hepatocellular carcinoma [6]. Although insulin resistance is usually associated with the development of type 2 diabetes, it can also be a feature of patients with type 1 diabetes [7]. Insulin resistance has been documented in type 1 diabetes and may contribute to the high risk for cardiovascular disease in this population [7]–[9]. In a recent review, it was stated that in type 1 diabetic population, an increased prevalence of obesity and insulin resistance often leads the development of nonalcoholic fatty liver diseases [10].

Zinc (Zn) is an essential trace element and plays a critical role in cellular integrity and biological functions in respect to cell division, growth, and development. Zn also acts as cofactor for many enzymes and proteins involved in the antioxidant, anti-inflammatory, and anti-apoptotic effects [11], [12]. The liver is important for the regulation of Zn homeostasis, while Zn is necessary for normal hepatic function [13]. Reduced hepatic Zn levels have been correlated with the impaired liver function and regeneration, and it also implicated in both acute and chronic liver disease states [14]–[16]. Zn supplementation offers a protection from acute and chronic liver injury in experimental animal models [17], [18], but these hepatoprotective properties have not been fully identified.

In the present study, therefore, we examined the effect of Zn deficiency on diabetes-induced hepatic pathogenic damage and apoptosis as well as possible mechanisms. To this end, we treated mice with multiple low-dose streptozotocin (MLD-STZ) to induce a type 1 diabetes. Zn deficiency was induced by chronic treatment with Zn chelator, N′N′N, N – tetrakis (2-pyridylemethyl) ethylenediamine (TPEN), as used in other studies [19], [20]. After diabetic and age-matched control mice were treated with and without TPEN for four months, hepatic pathological changes and cell death along with hepatic inflammation, oxidative damage, and insulin-related signaling pathways were examined.

## Materials and Methods

### Ethics Statement

This study was carried out in the strict accordance with the recommendations in the Guide for the Care and Use of Laboratory Animals of the National Institutes of Health. The protocol was approved by the Institutional Animal Care and Use Committee of the University of Louisville (IACUC #: 10155). All surgery was performed under anesthesia induced by intraperitoneal injection of 1.2% 2,2,2-Tribromoethanol (Avertin) at the dose of 0.2 ml/10 g body weight and all efforts were made to minimize suffering.

### Animal models

Male FVB mice, 8-weeks-old (18–22 of body weight), were obtained from Jackson Laboratory (Bar Harbor, Maine) and housed at 22°C with a 12∶12-h light-dark cycle and free access to rodent chow and tap water. Animals were kept under these conditions for 2 weeks before being used for the experiments.

Mice were given intraperitoneally MLD-STZ Sigma-Aldrich (St. Louis, MO, USA) at 50 mg/kg daily for 5 days. Five days after the last injection, blood glucose obtained from mouse tail-vein was measured with a SureStep complete blood glucose monitor (LifeScan, CA, USA). The blood glucose level ≥250 mg/dl was considered as hyperglycemia. Then hyperglycemic (diabetic, n = 12) and age-matched control (n = 14) mice were treated intraperitoneally with TPEN (Sigma, MO, USA) at 5 mg/kg daily or with vehicle for 4 months. The selection of TPEN to chronically deplete systemic Zn is based on several previous studies that have successfully used TPEN to lower the body's Zn levels without significant systemic toxic effects [19]. At the time of sacrifice, the liver was harvested for histopathology and protein studies.

### Measurement of hepatic Zn levels

Zn levels in the liver were measured by an atomic absorption spectrophotometer using air-acetylene flame after tissue was digested with nitric acid [21]. By this assay, total Zn in the tissue including free and protein-bound Zn was measured and expressed as µg/g wet tissue.

### Hepatic function biomarker detection

Serum plasma alanine aminotransferase (ALT) of these mice was measured using an ALT infinity enzymatic assay kit (Thermo Scientific, Waltham, MA).

### Histological examination

Liver tissue was fixed in 10% formalin and embedded in paraffin. Fixed liver tissues were cut into 5-µm slices. After being deparaffinized using xylene and ethanol dilutions and rehydration, tissue sections were stained with hematoxylin and eosin (H&E).

### Terminal deoxynucleotidyl transferase-mediated dUTP nick end labeling (TUNEL) assay

For TUNEL staining, slides were stained with the reagents supplied by ApopTag Peroxidase *In Situ* Apoptosis Detection Kit (Chemicon, Billerica, CA). Briefly, each slide was deparaffinized, rehydrated, and treated with proteinase K (20 mg/L) for 15 min. The endogenous peroxidase was inhibited with 3% hydrogen peroxide for 5 min, and then the slide was incubated with the TUNEL reaction mixture containing terminal deoxynucleotidyl transferase (TdT) and digoxigenin-11-dUTP for 1 h in a humidified chamber at 37°C. Then 3,3-diaminobenzidine chromogen was applied. Hematoxylin was used as counterstaining. For negative control, TdT was omitted from the reaction mixture. Apoptotic cell death was quantitatively analyzed by counting TUNEL positive cells selected randomly from ten fields at 40×. Results were presented as TUNEL positive cells per 10^3^ cells.

### Oil Red O staining for lipid accumulation

Cryosections from OCT-embedded tissue samples of the liver (10 µm thick) were fixed in 10% buffered formalin for 5 min. at room temperature, stained with Oil Red O for 1 h, washed with 10% isopropanol, and then counterstained with hematoxylin (DAKO, Carpinteria, CA) for 30 s. A Nikon microscope (Nikon, Melville, NY) was used to capture the Oil Red O – stained tissue sections at 40× magnification.

### Nuclei isolation

Hepatic nuclei were isolate using nuclei isolation kit (NUC- 201, Sigma, MO, USA). Briefly, 60 mg liver tissues from each mouse were homogenized for 45 sec. within 300 µl cold lysis buffer containing 1 µl dithiothreitol (DTT) and 0.1% Triton X-100. After that, 600 µl cold 1.8 mol/L Cushion Solution (Sucrose Cushion Solution: Sucrose Cushion Buffer: DDT = 900: 100: 1) was add to the lysis solution. The mixture was transferred to a new tube pre-loaded with 300 µl 1.8 mol/L Sucrose Cushion Solution followed by a centrifugation at 30,000× g for 45 min. The supernatant containing cytoplasmic component was saved for later analysis. Nuclei were visible as thin pellet at the bottom of tube.

### Western blotting assays

Western blotting assays were performed as described before [22]. Briefly, liver tissues and nuclei were homogenized in lysis buffer. Proteins were collected by centrifuging at 12,000 g at 4°C in a Beckman GS-6R centrifuge for 10 min. The protein concentration was measured by Bradford assay. The sample of total protein, cytoplasm protein or nuclear protein, diluted in loading buffer and heated at 95°C for 5 min, was subjected to electrophoresis on 10% SDS-PAGE gel. After electrophoresis of the gel and transformation of the proteins to nitrocellulose membrane, these membranes were rinsed briefly in tris-buffered saline, blocked in blocking buffer (5% milk and 0.5% BSA) for 1 h, and washed three times with tris-buffered saline containing 0.05% Tween 20 (TBST). The membranes were incubated with different primary antibodies overnight at 4°C, washed with TBST and incubated with secondary horseradish peroxidase–conjugated antibody for 1 h at room temperature. Antigen antibody complexes were then visualized using ECL kit (Amersham, Piscataway, NJ).

The primary antibodies used here include those against 3-nitrotyrosine (3-NT, 1∶1000, Chemicon), 4-hydroxynonenal (4-HNE, 1∶ 2000, Calbiochem, San Diego, CA), Tribbles homolog 3 (TRB3, 1∶1000, Calbiochem), inter-cellular adhesion molecule-1 (ICAM-1, 1∶ 500, Santa Cruz Biotechnology, Santa Cruz, CA), C/EBP homology protein (CHOP, 1∶ 500, Santa Cruz Biotechnology), plasminogen activator inhibitor type 1 (PAI-1, 1∶ 2000, BD Biosciences, Sparks, MD), Protein tyrosine phosphatase 1B (PTP1B, 1∶ 1000, BD Biosciences), nuclear factor-erythroid 2-related factor 2 (Nrf2, 1∶ 1000, Abcam, Cambridge, MA). Other primary antibodies, including tumor necrosis factor-α (TNF-α, 1∶500), total- and phospho-Akt (Ser473, 1∶500), total and phosphor-GSK-3β (1∶500), total- and phosphor-tensin homolog (PTEN, 1∶ 500), cleaved caspase-12 (1∶1000), Fyn (1∶1000), Bax and Bcl-2 (1∶ 1000) were purchased from Cell Signaling Technology (Danvers, MA).

### Triglyceride (TG) measurement

Liver tissues were homogenized in 1× PBS. Lipids were extracted with methanol: chloroform (1∶2), dried in an evaporating centrifuge, and resuspended in 1% Triton X-100. Colorimetric assessment of hepatic TG levels was carried out using Thermo scientific TG assay reagents (Thermo Fisher Scientific Inc.). Values were normalized to the protein concentration in homogenate before extraction, determined by the Bradford assay (Bio-Rad Laboratories, Hercules, CA).

### Cell culture and treatments

Human hepatocellular carcinoma cell (HepG2) line was maintained in Dulbecco's modified Eagle's medium (DMEM)/F12 supplemented with 10% fetal bovine serum from Invitrogen (Carlsbad, CA). In order to make Zn deficiency in the cultured cells, when cell populations reached 40–50% confluence, TPEN at 0.01–1 µM was added into the medium of some cultures for 30 h. After optimize the optimal dose of TPEN to induce apoptotic cell death, HepG2 cells were treat with the optimal dose of TPEN simultaneously with different doses of Zn (15–50 µM) for 30 h.

### Statistical analysis

Data were collected from repeated experiments and were presented as mean ± SD. One-way ANOVA was used to determine if difference exists. If so, a post hoc Turkey's test was used for analysis for the difference between groups, with Origin 7.5 laboratory data analysis and graphing software. Statistical significance was considered as *p*<0.05.

## Results

### Effect of TPEN and diabetes on hepatic Zn levels

**Figure 1:**
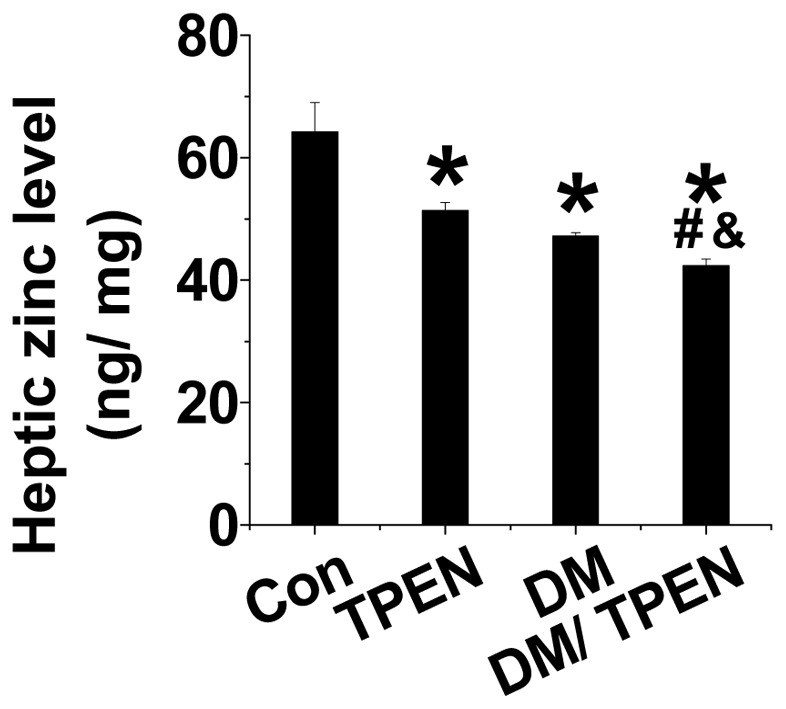
Effect of diabetes and TPEN treatment on hepatic zinc level. Diabetes was induced with MLD-STZ and treated with and without TPEN at 5 mg/kg daily for 4 months. Hepatic Zn levels were measured by atomic absorption spectrometer. Data are presented as mean ± SD (n = 6 at least in each group). DM: diabetes. * P<0.05 vs. control group; ^#^ P<0.05 vs. TPEN group; ^&^ P<0.05 vs. DM group.

Hyperglycemic and age-matched control mice were treated with and without TPEN for four months. Diabetes or TPEN treatment for 4 months mildly reduced hepatic Zn level (P<0.05, Fig. 1). TPEN treatment further decreased diabetic reduction of hepatic Zn level (Fig. 1), suggesting the induction of hepatic Zn deficiency in Diabetes and Diabetes/TPEN groups.

### Effects of Zn deficiency on diabetes-induced hepatic damage and steatosis

**Figure 2:**
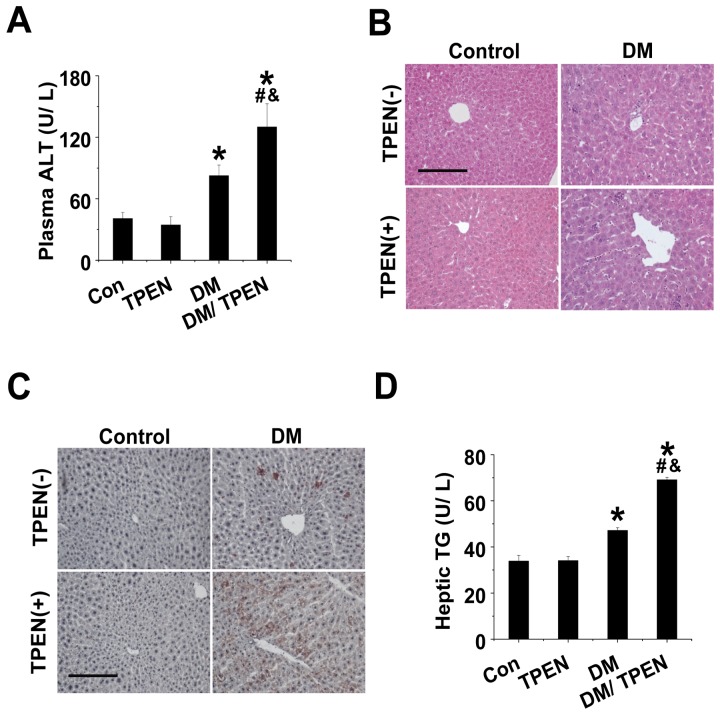
Diabetes and TPEN treatment induced the ALT increase in the plasma and also morphological changes and lipid accumulation in the liver. Plasma ALT levels were measured by infinity TM ALT detection kit (A). Hepatic morphological changes were examined microscopically with H&E staining (40×) (B). Hepatic lipid accumulation (C) and TG levels (D) were examined by Oil Red O staining (40×) and by TG reagent, respectively. Data are presented as mean ± SD (n = 6 at least in each group). DM: diabetes. * P<0.05 vs. control group; ^#^ P<0.05 vs. TPEN group; ^&^ P<0.05 vs. DM group.

As one of measurements for hepatic damage, serum ALT level was not changed in TPEN-treated non-diabetic group, but significantly increased in diabetic group, which was further enhanced by TPEN treatment in diabetic mice (Fig. 2A).

Liver pathology with H&E staining is presented in Fig. 2B. The hepatic cell structure in control group was normal and clear without inflammation and necrosis. In TPEN treatment group, a few inflammatory cells were observed with the same cell structure as those seen in control group. However, diabetes increased hepatic damage with obviously necrotic and/or inflammatory foci. In the liver of Diabetes/TPEN group, the morphological change was more severe with more inflammatory and/or necrotic foci as compared to the liver of Diabetes group.

Examination of hepatic lipid accumulation status with Oil red O staining revealed that no lipid accumulation was observed in control or TPEN treatment group; however, significant lipid accumulation was observed in Diabetes group, which was further increased in Diabetes/TPEN group (Fig. 2C). TG measurement with ELISA showed the significant increase of hepatic TG levels in Diabetes/TPEN compared to Diabetes or TPEN alone (Fig. 2D).

### Effects of Zn deficiency on diabetes-induced hepatic apoptotic cell death

**Figure 3:**
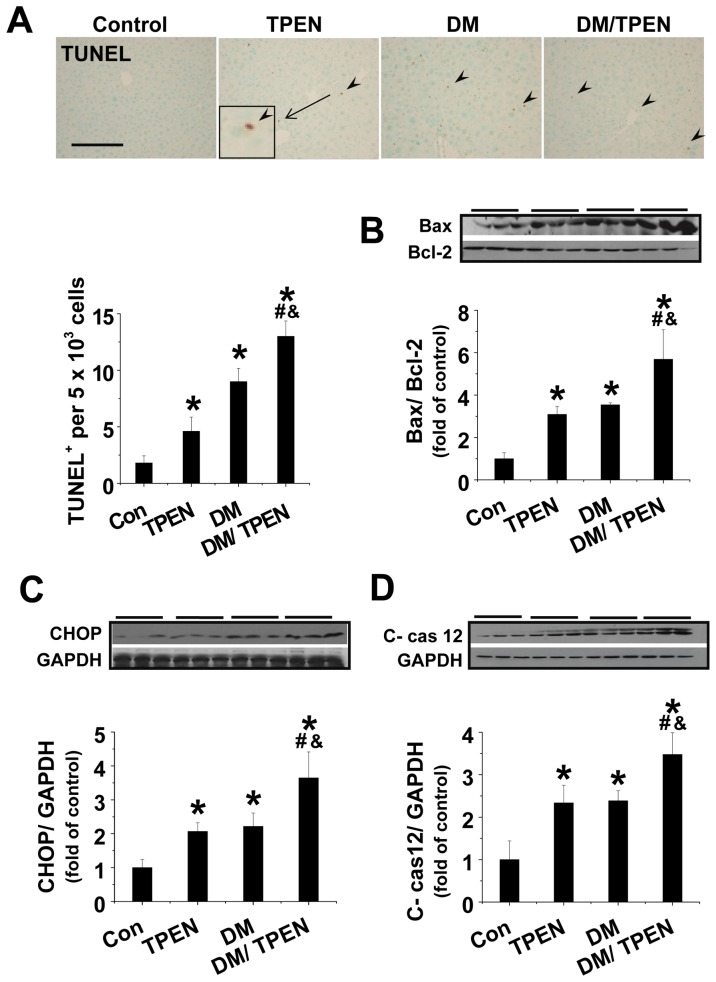
Diabetes and TPEN treatment induced hepatic apoptosis, which was related to hepatic ER stress. Apoptotic cell death was examined by TUNEL staining, followed by the quantitative analysis of the positive cells (A). The expression of Bax and Bcl-2 were detected by Western blotting assay and the ratio of Bax/Bcl-2 was present (B). ER stress-associated cell death was examined by Western blotting for the expression of CHOP (C) and cleaved caspase 12 (D). Data are presented as mean ± SD (n = 6 at least in each group). DM: diabetes. * P<0.05 vs. control group; ^#^ P<0.05 vs. TPEN group; ^&^ P<0.05 vs. DM group.

By examination of hepatic apoptosis with TUNEL staining, an increase of TUNEL positive cells was mildly and significantly evident in the liver of TPEN and Diabetes groups, respectively. Diabetes/TPEN group showed a synergistic outcome in respect to the apoptotic effect (Fig. 3A).

Analysis of Bax/Bcl-2 ratio as one of mitochondrial cell death pathway disclosed a synergistic increase in Bax/Bcl-2 ratio in the liver of Diabetes/TPEN as compared to Diabetes and TPEN alone (Fig. 3B).

Next whether Zn-deficiency enhanced diabetes-induced hepatic cell death is associated with endoplasmic reticulum (ER) stress related cell death pathway was examined by Western blotting of CHOP expression and caspase-12 cleavage. Both Zn deficiency and diabetes were found to significantly increase CHOP expression (Fig. 3C) and caspase-12 activation (Fig. 3D), suggesting that Zn deficiency or diabetes induced ER hepatic cell death is associated with ER stress. Diabetes/TPEN showed a synergistic effect on these changes.

### Diabetes-induced hepatic inflammation and oxidative damage, which were exacerbated by Zn deficiency

**Figure 4:**
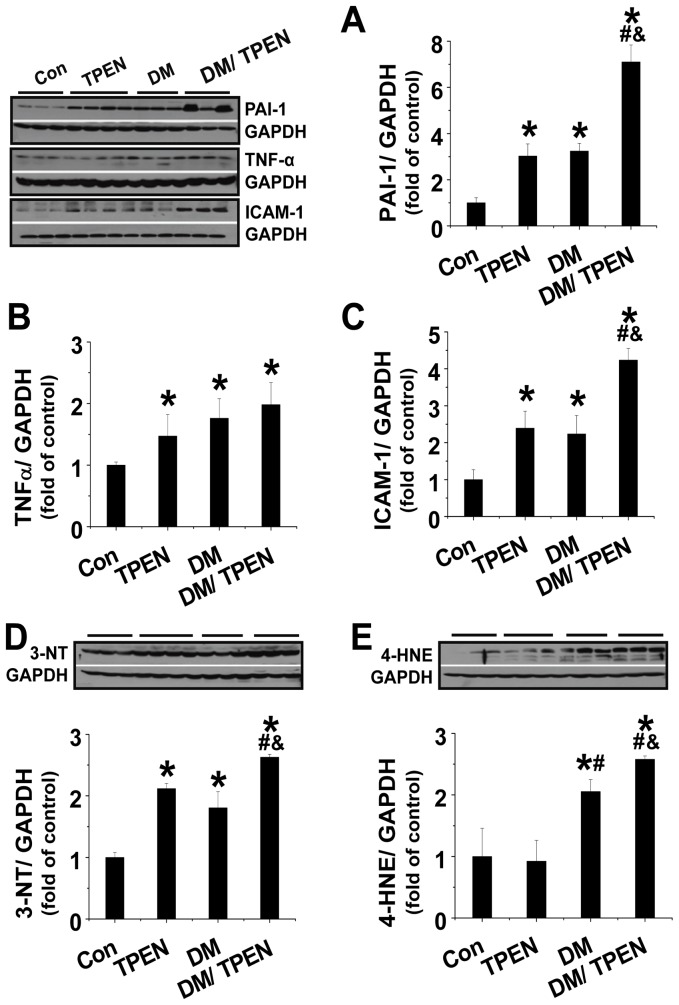
Effects of diabetes and TPEN on hepatic inflammation and oxidative damage. Hepatic expression of inflammatory factors, including PAI-1 (A), TNF-α (B), and ICAM-1 (C) was examined by Western blotting. Hepatic oxidative damage was examined by Western blotting assay for the expression of 3-NT as an index of protein nitration (D) and 4-HNE as an index of lipid peroxidation (E). Data are presented as mean ± SD (n = 6 at least in each group). DM: diabetes. * P<0.05 vs. control group; ^#^ P<0.05 vs. TPEN group; ^&^ P<0.05 vs. DM group.

In respect that both diabetes and Zn deficiency cause inflammation, we examined whether the exacerbation of diabetes-induced hepatic cell death and steatosis by Zn deficiency is associated with the exacerbation of diabetic inflammatory response and oxidative stress. Western blotting revealed that both diabetes and Zn deficiency significantly up-regulated the expression of PAI-1(Fig. 4A), TNF-α (Fig. 4B), and ICAM-1(Fig. 4C). Treatment of diabetic mice with TPEN enhanced the expression of these inflammatory cytokines induced by diabetes.

Inflammatory response often causes, or is accompanied with, oxidative stress and damage; therefore, whether there was an increase of hepatic oxidative stress and damage was examined by protein nitration and lipid peroxidation with Western blotting of 3-NT and 4-HNE, respectively. There was a significant increase of 3-NT expression in both TPEN treatment and diabetes groups. Treatment of diabetes with TPEN induced a synergetic effect on the expression 3-NT expression (Fig. 4D). A significant increase of 4-HNE was also seen in the liver of diabetic mice, but not in the liver of TPEN-treated mice. A further increased hepatic accumulation of 4-HNE was observed in Diabetes/TPEN group compared to Diabetes alone (Fig. 4E). These results indicated Zn deficiency significantly enhanced the oxidative and nitrosative damage induced by diabetes.

### Mechanistic study on the exacerbation of diabetes-induced hepatic injury by Zn deficiency: the critical role of Nrf2

**Figure 5:**
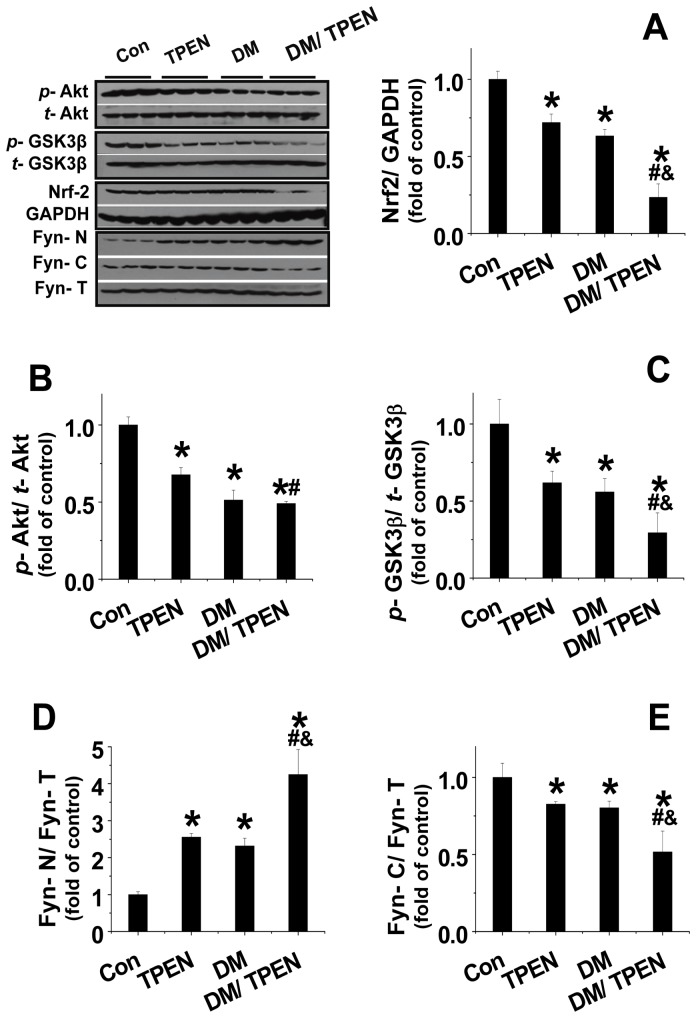
Diabetes and TPEN treatment decreased the expression of Nrf2 that was associated with decreased Akt and GSK-3β phosphorylation and nuclear accumulation of Fyn. The expression of Nrf2 (A) and total and phosphorylated Akt (B) and GSK3β (C) were examined with Western blotting. The expression of Fyn in nuclei, cytosol, and total tissues were measured by Western blotting, for which the ratios of Fyn-N/Fyn-T (D) and Fyn-C/Fyn-T (E) were presented. Data are presented as mean ± SD (n = 6 at least in each group). DM: diabetes; Fyn- N: Nuclear Fyn; Fyn- C: cytosolic Fyn; Fyn- T: Total tissue Fyn. * P<0.05 vs. control group; ^#^ P<0.05 vs. TPEN group; ^&^ P<0.05 vs. DM group.

Nrf2 is one of the most important cellular defense mechanism against oxidative stress. In response to oxidative stress, Nrf2 can translocate into nucleus and induce transcription of genes encoding various protective antioxidants [23], [24]. Therefore, whether the increased oxidative and nitrosative stress is related to down-regulation of Nrf2 expression in the liver was examined. We found that both Zn deficiency and diabetes significantly decreased hepatic Nrf2 expression and there was a synergistic effect of Zn deficiency and diabetes together on the down-regulation of Nrf2 expression (Fig. 5A).

It was reported recently that Nrf2 was negatively regulated by GSK-3β via its phosphorylation of Fyn that stimulates export of Nrf2 from nuclear to cytosol where it was degraded. We found that although Zn deficiency or diabetes alone slightly decreased (P<0.05), diabetes with Zn deficiency further decreased, the phosphorylation of Akt (Fig. 5B) and GSK-3β (Fig. 5C). The down-regulation of Akt and GSK-3β phosphorylation was accompanied with an increase of nuclear Fyn accumulation (Fig. 5D) and a decrease of cytosol Fyn accumulation (Fig. 5E).

**Figure 6:**
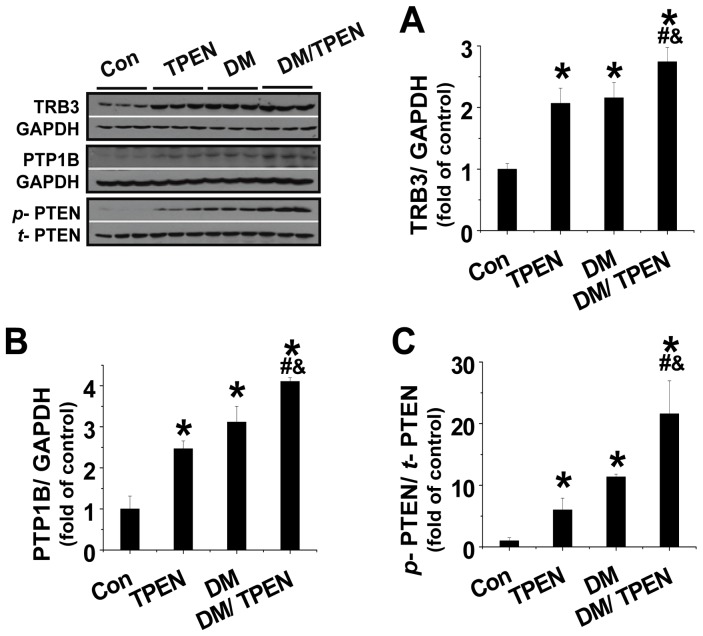
Diabetes and TPEN treatment activated Akt negative regulators. Hepatic expressions of TRB3 (A), PTP1B (B), and phosphorylated PTEN (C) were examined by Western blotting. Data are presented as mean ± SD (n = 6 at least in each group). DM: diabetes. * P<0.05 vs. control group; ^#^ P<0.05 vs. TPEN group; ^&^ P<0.05 vs. DM group.

To explore how diabetes decreases, and Zn deficiency enhances diabetic decrease in, the phosphorylation of Akt and GSK-3β, the expression or phosphorylation of Akt negative regulators TRB3 (Fig. 6A), PTEN (Fig. 6B), and TPT1B (Fig. 6C) was examined. Results showed that diabetes or Zn deficiency (TPEN) significantly increased TRB3 and PTP1B expression, and Diabetes/TPEN further increased their expressions (Fig. 6A,B). Diabetes or Zn deficiency also increased, and Diabetes/TPEN further increased, the phosphorylation of PTEN (Fig. 6C).

### TPEN-induced hepatic cell death was rescued by supplementation of Zn

**Figure 7:**
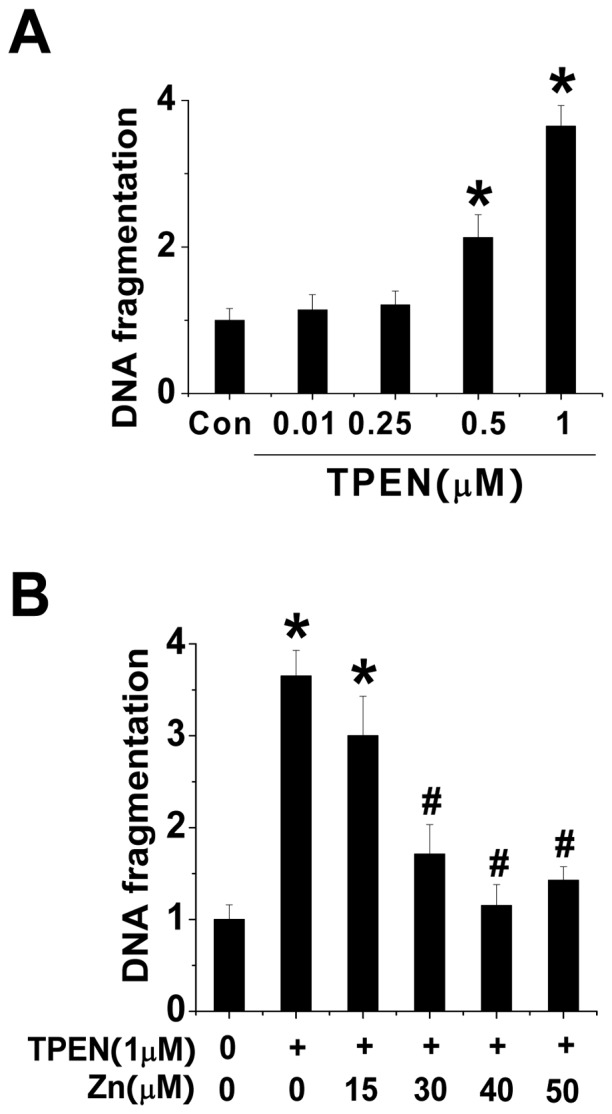
TPEN induced apoptosis in HepG2 cells is prevented by supplementation of Zn. HepG2 cells were treated by indicated concentrations of TPEN for 30 h and then the cell death was detected by DNA fragmentation assay (A). HepG2 cells were also treated with 1 µM TPEN in the absence or presence of Zn at indicated concentrations for 30 h and then the cell death was detected by DNA fragmentation assay (B). Experiments were repeated three times at least and the data are presented as mean ± SD. * P<0.05 vs. control group; ^#^ P<0.05 vs. TPEN group.

To investigate whether TPEN increased hepatic damage in the animal model is due to Zn deficiency, rather other TPEN's direct toxicity, we have performed an in vitro study, in which HepG2 cells were exposed to TPEN at different doses (0.1–1.0 µM) for 30 h to induce the cell death. We found that 30-h exposure to TPEN at 0.5 and 1.0 µM induced a significant increase in apoptotic cell death, shown by DNA fragmentation (Fig. 7A). We also found that when cells were exposed to 1.0 µM TPEN with and without Zn, the addition of Zn at 30–50 µM in the medium can completely rescue TPEN-induced apoptotic effect (Fig. 7B), suggesting that TPEN-induced apoptotic effect is due to its Zn chelation effect, instead of its direct toxic effect.

## Discussion

**Figure 8:**
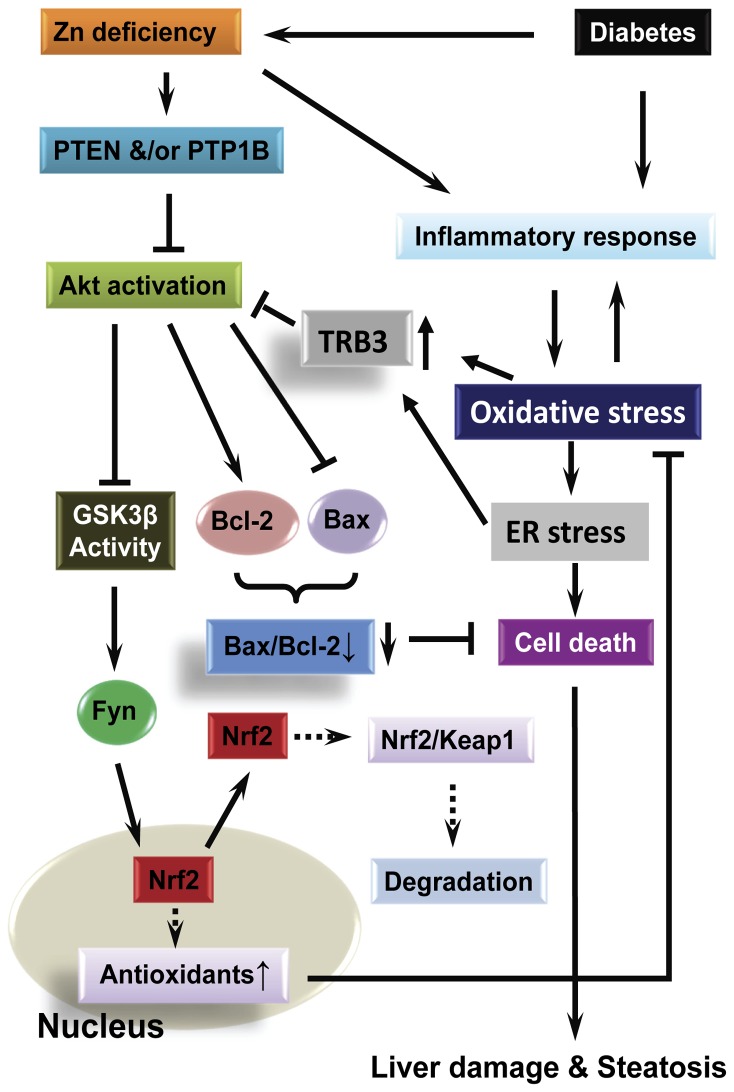
Possible mechanism for hepatic damage induced by diabetes and Zn deficiency. Both Zn deficiency-induced PTEN/PTP1B activation and diabetes-increased TRB3 expression via induction of oxidative stresses and ER stress inhibit the activation of Akt, which in turn increase GSK3β activity, leading to Fyn-nuclear accumulation that stimulates Nrf2 exporting to cytosol where to be degraded. Down-regulation of Nrf2 activity leads to the decrease in antioxidants, which cause hepatic oxidative stress, inflammation, cell death, damage, and steatosis.

In the present study, we have demonstrated the hepatic injury, including inflammatory response, lipid accumulation, and hepatic cell death along with the increased serum hepatic enzyme, in the type 1 diabetic animals. Diabetes-induced hepatic injury was exacerbated by Zn deficiency induced by chronic treatment with TPEN. Nrf2 as an important transcription factor was found to be decreased in the liver of diabetic and Zn deficient groups, and further decreased in the liver of diabetic mice with Zn deficiency (Diabetes/TPEN). We also found that Zn deficiency exacerbated diabetic inhibition of Akt and GSK-3β phosphorylation along with an up-regulation of Akt negative regulators. The decreased phosphorylation of GSK-3β is accompanied with a significant increase in nuclear accumulation and decrease in cytosolic accumulation of Fyn. Therefore, we concluded that Zn deficiency significantly exacerbates diabetes-induced hepatic damage, which is likely because Zn deficiency exacerbates diabetic down-regulation of Nrf2 expression and function by up-regulation of Akt negative regulators. Up-regulated Akt negative regulators down-regulate the phosphorylation of Akt and GSK-3β, leading to Fyn nuclear translocation that exports Nrf2 to cytosol where being degraded, as shown in Fig. 8.

Although TPEN is a multi-heavy metal chelator, it has very high affinity for Zn and iron, and very low affinities for other metals such as calcium and magnesium [25]. Zn is the most abundant trace metal in the human body and has long been known to be an essential element for cell metabolism, anti-oxidation and survival [26], [27]. Zn deficiency will lead to cell metabolic dysfunction and cell death by induction of caspase activation [28], [29]. In the present study we showed that the hepatic Zn level was decrease in TPEN-treated normal mice and further decrease in TPEN-treated diabetic mice (Fig. 1). Therefore the hepatic toxicity of TPEN-treatment in normal and diabetic mice should be mediated by Zn deficiency. To further confirm our speculation, we further provided experimental evidence that TPEN-treated hepatic cells also showed the induction of apoptotic cell death in a dose-dependent manner (Fig. 7A) and the apoptotic effect of TPEN in the hepatic cells was able to be completely rescued by supplementation of Zn at 30–50 µM, but not at lower level such as 15 µM (Fig. 7B). The in vitro study suggests that the apoptotic effect of TPEN treatment is mediated by its chelation of Zn, rather than its direct toxicity.

Type 1 diabetic patients with the liver disease often have systemic increases of inflammatory cytokines such as TNF-α [30], [31]. Zn deficiency also induces systemic inflammatory response [32], [33] and hepatic injury [14]–[16]. Therefore, we assumed that Zn deficiency in diabetic patients might exacerbate hepatic injury. In support of our hypothesis, we demonstrated here that Zn deficiency significantly exacerbated diabetes-induced hepatic inflammation, oxidative stress, lipid accumulation, and hepatic cell death along with the increased serum hepatic enzyme (Figs. 2,3,4). Our finding is consistent with a previous study that showed the exacerbation of carbon tetrachloride hepatic toxicity by Zn deficiency [34].

Endoplasmic reticulum (ER) stress was found to play a critical role in diabetes pathogenesis and diabetes-induced testicular and cardiac apoptosis [22], [35]. To date, however, there was no report whether ER stress also plays certain role in diabetes-induced hepatic cell death. In fact, hepatocytes contain abundant ER that is essential for protein metabolism and stress signaling. Hepatic cells cope with ER stress by an adaptive or protective response, termed unfolded protein response (UPR). UPR includes both the enhancement of protein folding and degrading in the ER and the down-regulation of overall protein synthesis. When the UPR to ER stress is insufficient, the ER stress response unleashes pathological consequences, including hepatic fat accumulation, inflammation and cell death, which can lead to the liver disease or worsen other causes-induced liver diseases [36]. Consistent with these early observations, here we demonstrated the induction of ER stress in the liver of diabetic mice (Fig. 3C,D), shown by increased CHOP and caspase-12 cleavage, which was worsened in the diabetic mice with Zn deficiency. These data suggest that either diabetes or Zn deficiency induces the hepatic ER stress-related cell death and two pathogeneses together caused a synergetic effect on the ER stress and cell death.

There were several previous studies that have demonstrated the negative regulation of Nrf2 by Fyn via its forcing Nrf2 exportation from nucleus to cytosol where Nrf2 binds to Keap1 for its degradation. Since GSK-3β controls Fyn translocation into nucleus, the inactivation of GSK-3β by its phosphorylation results in a less nuclear accumulation of Fyn [37], [38]. Zn has been reported to negatively regulate Akt negative regulators PTP1B [39], [40] and PTEN [41]. Therefore, we assume that the exacerbation of hepatic injury by Zn deficiency may be because Zn deficiency loses its inhibition of PTP1B and PTEN, leading to the inhibition by these two negative regulators of Akt phosphorylation and consequently down-regulation of GSK-3β phosphorylation, which will increase Fyn nuclear accumulation to export Nrf2 into cytosol, as shown in Fig. 8.

TRB3 is a novel ER stress-inducible protein [42], [43]. Here we showed the increases in CHOP expression and caspase-12 activation in the liver of Zn deficiency and diabetes groups at a similar level but a synergistic increase in the liver of diabetes with Zn deficiency (Fig. 3D,E). Similarly there was also a similar level of increase of TRB3 expression in the liver of Zn deficiency and diabetes alone groups, but there was a synergistic increase of TRB3 expression in the liver of Diabetes/TPEN group. Therefore, we assume that due to down-regulation of Nrf2 function, less transcriptional expression of multiple antioxidants would result in a further increase in diabetic oxidative stress, which directly or indirectly via ER stress up-regulates TRB3 that directly inhibits Akt function, as illustrated in Fig. 8.

In summary, we have explored here the effect of Zn deficiency on diabetic liver injury in the type 1 diabetes mouse model. We found that Zn deficiency exacerbated diabetes-induced hepatic oxidative damage, inflammation, and cell death, through down-regulation of Nrf2 expression and transcription. In respect that patients with diabetes often have some levels of Zn deficiency that may be partially due to increased urinary Zn excretion and partially due to restriction of certain food intakes [44], [45], and about 12% of Americans do not consume the average requirement for Zn so that they could be at risk for marginal Zn deficiency [46], [47], we would like to draw the attention of patients with diabetes that proper intake of Zn may be important for the prevention of their diabetic complications, including diabetic liver injury.
